# Neonatal Adverse Outcomes of Induction and Expectant Management in Fetal Growth Restriction: A Systematic Review and Meta-Analysis

**DOI:** 10.3389/fped.2020.558000

**Published:** 2020-10-30

**Authors:** Ting Li, Yixiao Wang, Zhijing Miao, Yu Lin, Xiang Yu, Kaipeng Xie, Hongjuan Ding

**Affiliations:** Nanjing Maternity and Child Health Care Hospital, Women's Hospital of Nanjing Medical University, Nanjing, China

**Keywords:** fetal growth restriction (FGR), induction, expectant management, risk factors, meta-analysis

## Abstract

**Background and Objective:** Fetal growth restriction (FGR) is a pathological condition in which the fetus cannot reach its expected growth potential. When it is diagnosed as a suspected FGR, it remains an unsolved problem whether to direct induction or continue expectant management. To effectively reduce the incidence of neonatal adverse outcomes, we aimed to evaluate whether either method was associated with a lower incidence of neonatal adverse outcomes.

**Methods:** We searched the relevant literature through the PubMed, Web of Science, and Cochrane Library from inception to January 10, 2020. We defined induction as the experimental group and expectant management as the control group. Pooled odds ratios (ORs) with 95% confidence intervals (CIs) were calculated using random-effects models owing to heterogeneity. Furthermore, we conducted a sensitivity analysis to explore the robustness of the included literature. We used the Newcastle-Ottawa scale (NOS) to evaluate the quality of the available studies. We applied the funnel plot to describe the publication bias. Additionally, subgroup analysis based on the study method, sample size, area, NOS score, Apgar score <7 at 5 min, definition of suspected FGR, severity, and neonatal adverse outcomes were performed to further evaluate the differences between the induction and expectant management.

**Results:** Our study included a total of eight articles with 6,706 patients, which consisted of four randomized controlled trials (RCTs), three retrospective cohort studies, and one prospective cohort study. The total pooled OR and 95% CI between the induction group and the expected management group was 1.38 (95% CI, 0.84–2.28) in the random model. The heterogeneity was *I*^2^ = 84%, *P* < 0.01. The sensitivity analysis showed that the neonatal adverse outcomes of induction vs. expectant management still presented similar outcomes after omitting of any one of these studies. The funnel plot and linear regression equation showed that there was no publication bias in our study (*P* = 0.75). Subgroup analysis showed that induction increased the neonatal adverse outcome risks of hypoglycemia and respiratory insufficiency (OR_neonatal hypoglycaemia_ = 8.76, 95% CI: 2.57–29.90; OR_respiratory insufficiency_ = 1.74, 95% CI: 1.35–2.24, respectively). However, no significant differences were observed based on the other subgroups (all *P* > 0.05).

**Conclusion:** Regardless of induction or expectant management of a suspected FGR, the neonatal adverse outcomes showed no obvious differences. More studies should be conducted and confounding factors should be taken into consideration to elucidate the differential outcomes of the two approaches for suspected FGR.

## Introduction

Fetal growth restriction (FGR) is a pathological condition in which the fetus does not reach its expected growth potential (1). The pregnant maternal morbidity of FGR is ~5–10% (2). According to relevant statistics, neonatal mortality of FGR is 2–4 times that of normal newborns, which is the second most common cause of perinatal fetal death (3). Those with FGR were more prone to have complications in infancy and later stages, such as neonatal hypoglycemia, respiratory distress, and neurological, cardiovascular, and metabolic diseases, etc. (4–6). A systematic meta-analysis showed that children with an early-onset FGR were 34% more likely to have respiratory distress, 30% more likely to have sepsis, and 12% more likely to have a cognitive impairment or cerebral palsy (6).

However, until now, the definition of FGR has remained controversial. Differences among FGR, small for gestational age (SGA), and intrauterine growth restriction (IUGR) are not clear. Generally, the estimated fetal weight (EFW) <10th centile is accepted by most institutions. FGR can be classified as early-onset (<32 w) and late-onset (≥32 w), with the latter accounting for approximately about 70% of the cases (7). Existing studies have indicated that the diagnosis of FGR is based on the Doppler ultrasonography and EFW. The main parameters of Doppler ultrasonography are the umbilical artery (UA) pulse index (PI) and uterine artery (UTA) PI (7). Recently, the cerebral–placental ratio (CPR) and cerebral–placental–uterine ratio (CPUR) were identified as novel predictors, and at a 90% specificity, a low CPUR had sensitivities of 50% for birthweight <10th centile (8). Additionally, maternal serum biomarkers are novel approaches to screening the suspected FGR (PIGF, sFlt-1), but the specific effectiveness needs more studies for verification (7, 9). A study showed that 82% of stillbirths with FGR were not detected in the prenatal period (9) so accurate identification is important. Ultrasonic Doppler is the main means of monitoring. Doppler can reasonably and accurately reflect the condition of FGR in the early period, providing a basis for improving the clinical management of FGR, but to the choice of follow-up treatment remains a problem (10).

In recent years, there is still no consensus on the appropriate delivery timing for FGR. The etiology of FGR includes the infant, maternal, umbilical cord, and placental factors. With FGR, a pathological condition, the intrauterine environment is not suitable for the fetus to continue to grow in the mother. If gestational age is prolonged, it may increase the risk of hypoxia, acidosis, or even death, however, immediate induction also involves risks of neurological complications (11, 12). Nevertheless, appropriate delivery timing is vital to the FGR outcomes and it can also decrease the fetal and neonatal morbidity and mortality (13, 14). However, one RCT study demonstrated that immediate delivery and expectant treatment had no significant impact on the neonatal outcomes of FGR (15). When it is diagnosed as a suspected FGR, the problem of choosing direct induction or continuing expectant management, which can effectively reduce the incidence of neonatal adverse outcomes, remains disputed. We aimed to evaluate which method could have a lower incidence of neonatal adverse outcomes.

## Methods

This systematic review abided by the Preferred Reporting Items for Systematic Reviews and Meta-Analyses (PRISMA) guidelines (16).

### Literature Search

We searched for the relevant literature through the PubMed, Web of Science, and Cochrane Library from inception to January 10, 2020. The search standard included the MeSH (medical subject heading) terms, entry terms, and keywords. We did not restrict the language. The details of the search process are depicted in Appendix 1 (Supplementary Material). Two authors independently collected and integrated the data.

### Eligibility Criteria

We selected articles on the basis of the database searches and applied the EndNote X9 to clear the duplicate articles, then we browsed through the titles and abstracts to exclude the unrelated articles. Reviews, posters, commentaries, studies with, incomplete data or a lack of data, and duplicate citations were also excluded. Randomized controlled trials (RCTs) and cohort studies were included in our analysis. We added additional studies based on the references in related articles. We selected the articles with a suspected FGR (SGA, IUGR) at the late preterm and at term, and compared the adverse neonatal outcomes between the induction and expectant management. The definition of FGR was EFW or abdominal circumference (AC) <10th centile or ≤1.5 standard deviation (SD) for gestational age (GA) and gender or having an abnormal UA Doppler waveform. Adverse neonatal outcomes included stillbirth, fetal or newborn death, neonatal intensive care unit (NICU), arterial pH <7.05, Apgar score <7 at 5 min, hypoglycemia, hyperbilirubinemia, respiratory insufficiency, and neonatal sepsis.

### Data Extraction and Study Quality Assessment

The first author's name, study methods, year of research, sample size, area, gestational age, definition of FGR (SGA, IUGR), number of neonatal adverse outcomes in the induction group and expectant management group, and neonatal adverse outcomes were obtained from the included articles. The extracted data provided effective information to construct 2 × 2 tables. Two authors independently abstracted the information and disagreements were resolved by the corresponding author. We conducted a sensitivity analysis to explore the robustness of the included literature. The study quality assessment was based on the Newcastle-Ottawa scale (NOS) (17–19). Using this protocol, the maximum score of each study was nine. Studies with a minimum score >7 were regarded as high-quality articles (20). Two authors independently gave a mark on each study and decided whether it was eligible for inclusion in our meta-analysis, and if any controversy existed in the decision, the corresponding author joined the discussion. Additionally, we applied the funnel plot and linear regression equation to describe the publication bias. Subgroup analysis based on the study method, sample size, area, NOS score, Apgar score <7 at 5 min, definition of a suspected FGR, severity, and neonatal adverse outcomes were performed to further evaluate the significance between the induction and expectant management.

### Statistical Analysis

We defined induction as the experimental group and expectant management as the control group and then compared the neonatal adverse outcomes between them. Additionally, we selected study methods, sample size (≤500, >500), area, NOS score (≤7, >7), Apgar score <7 at 5 min, definition of a suspected FGR, severity, and neonatal adverse outcomes to conduct the subgroup analysis. Article risk evaluation was performed in the Review Manager (RevMan) version 5.3. All other data were analyzed via the R version 16. Publication bias was evaluated by the funnel plots and linear regression equations. Forest plots were constructed to obtain pooled ORs and 95% CIs. If *I*^2^ < 50%, the fixed effects model was performed to calculate the pooled effect estimates. If *I*^2^ ≥ 50%, the random effects model was applied. The cut-off value of *P* < 0.05 was defined as statistically significant.

## Results

### Study Selection

**Figure 1:**
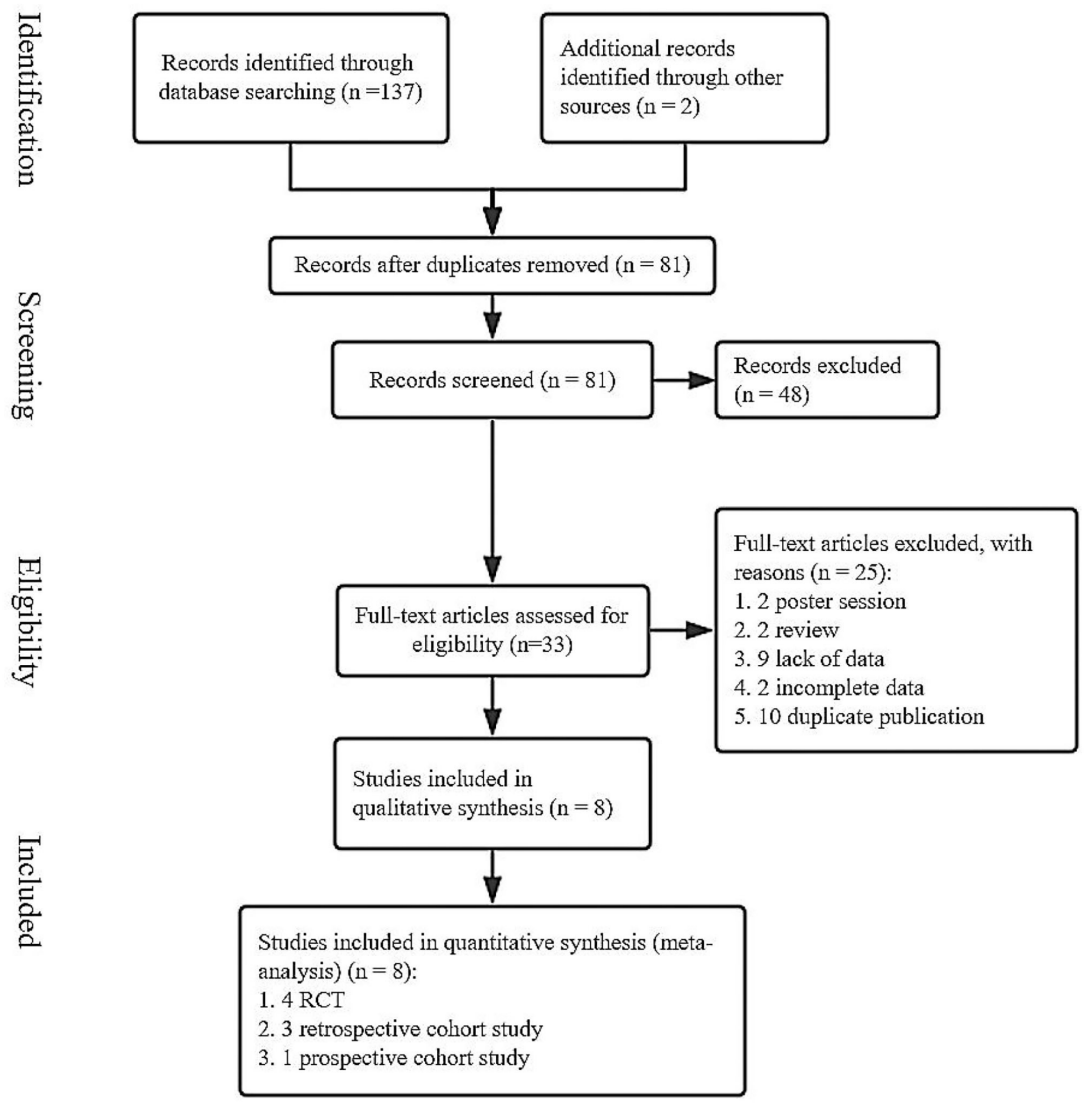
A flow chart of screened studies on induction vs. expectant management of neonatal adverse outcomes.

We searched for the relevant literature through the PubMed, Web of Science, and Cochrane Library from inception to January 10, 2020. A total of 137 studies were obtained and two studies were obtained via relevant references (Figure 1). After removing duplicate articles, 81 articles are remained. Then, the irrelevant and data–deficient articles were eliminated by browsing the titles, abstracts, and full-text. Finally, we included eight articles in this meta-analysis (15, 21–27).

### Study Characteristics

The main characteristics of the included studies were depicted in Table 1. This meta-analysis included eight articles with 6,706 patients, which consisted of four randomized controlled trials (RCTs), three retrospective cohort studies and one prospective cohort study. The publication time of these articles ranged from 2006–2019. Among these articles, the study areas included the Netherlands for three studies; Israel, three; Japan, one; and The United Kingdom, one. The minimum sample size was 33 and the maximum sample size was 2,378. For the eight studies, one study included pregnant women with a GA ranging from 24 to 36 weeks, one was between 34 and 38 weeks, and all the other studies started with pregnant women whose GA ≥36 weeks. For the diagnosis of a suspected FGR, most of these studies adopted the EFW or AC <10th centile for GA and/or gender, and one study used an EFW ≤1.5 SD for GA. Only one study applied a criteria involving an abnormal umbilical artery Doppler waveforms. Apgar score <7 at 5 min was used as one of the neonatal adverse outcomes in six studies. One study included only neonatal death and disability, while another study recorded only the adverse outcome of newborns with an Apgar score <7 at 5 min. More than four kinds of adverse neonatal outcomes were included in other studies, but the evaluation criteria used in these studies were slightly different.

**Table 1:** Characteristics of the included publications.

**References**	**Study methods**	**Area**	***N***	**Gestational age (w)**	**Definition of suspected FGR**	**Induction vs. expectant management (*N*)**	**Neonatal adverse outcomes (induction vs. expectant management, *N*)**	**Neonatal adverse outcomes criteria**
van den Hove et al. (26)	Prospective cohort study	Netherlands	33	>37	AC <10th centile or a declining FAC curve for GA	16 vs. 17	8 vs. 6	a, b, c, d, e, f
Boers et al. (15)	RCT	Netherlands	650	Range, 36–41	AC or EFW <10th centile or flattening of the growth curve for GA	321 vs. 329	17 vs. 20	g, h, i, j
Walker et al. (27)	RCT	The United Kingdom	302	Range, 24–36	UA Doppler waveform is abnormal	153 vs. 149	21 vs. 25	g, k
Shavit et al. (25)	Retrospective cohort study	Israel	669	≥37	EFW <10th centile for GA	170 vs. 499	0 vs. 3	h
Ofir et al. (23)	RCT	Israel	2,378	Range, 37–39	EFW <10th centile for GA and gender	445 vs. 1,933	85 vs. 210	a, c, f, h, l, m, n, o, p, q, r, s, t
Rabinovich et al. (2018)	Retrospective cohort study	Israel	2,232	Range, 34–38	EFW <10th centile for GA	1,428 vs. 804	290 vs. 173	a, g, h, u, v, w, x
Hidaka et al. (22)	Retrospective cohort study	Japan	150	Equal to 37	EFW ≤ 1.5 SD for GA	73 vs. 77	45 vs. 14	h, j, m, p
van Wyk et al. (21)	RCT	Netherlands	292	>36	AC or EFW <10th centile or flattening of the growth curve for GA	158 vs. 134	7 vs. 7	h, i, j

*RCT, randomized controlled trial; N, number; w, week; FGR, fetal growth restriction; AC, abdominal circumference; FAC, fetal abdominal circumference; GA, gestational age; EFW, estimated fetal weight; UA, umbilical artery; SD, standard deviation; a, hypoglycaemia; b, hypothermia; c, thrombocytopenia; d, hyponatremia; e, oxygen administration; f, infection; g, neonatal death; h, Apgar score <7 at 5 min; i, umbilical artery pH <7.05; j, NICU; k, disability; l, necrotizing enterocolitis; m, respiratory insufficiency; n, neurological complications; o, blood transfusion; p, hyperbilirubinemia; q, phototherapy; r, meconium aspiration syndrome; s, birth trauma; t, neonatal resuscitation; u, fetal distress; v, stillbirth; w, neonatal sepsis; x, prematurity complications*.

### Total Pooled Effect

**Figure 2:**
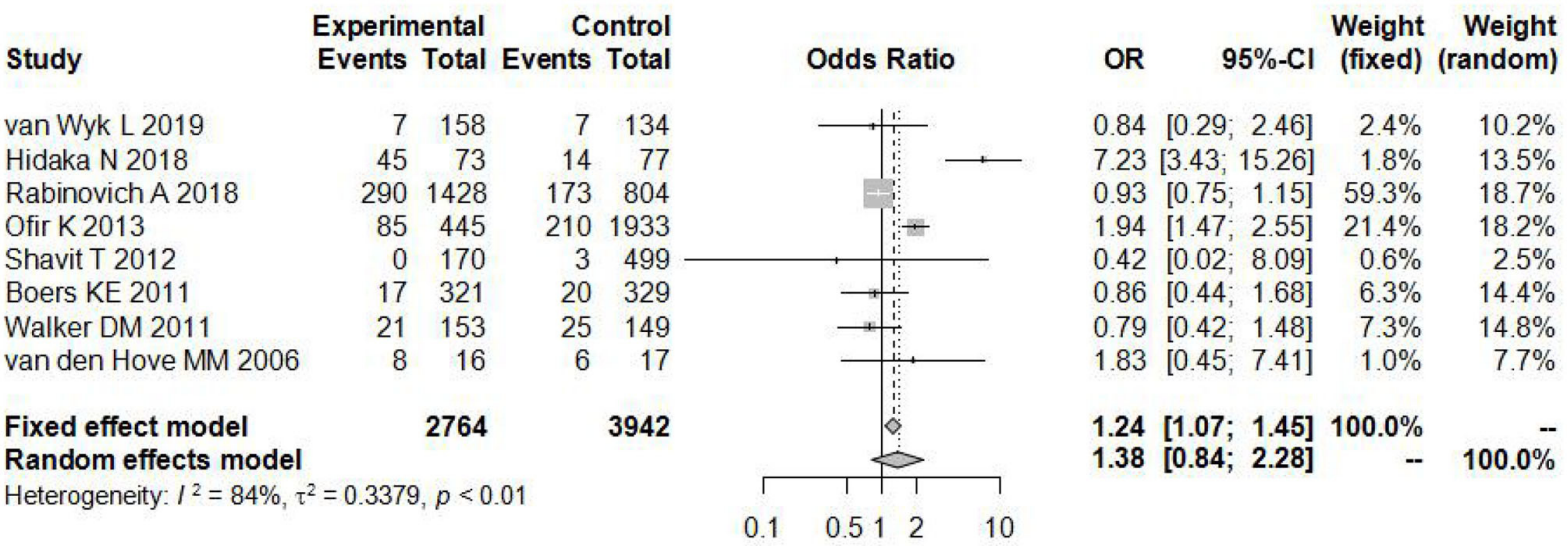
Individual study odds ratio (95% CI) for neonatal adverse outcomes of induction vs. expectant management.

As shown in Figure 2, there was a significant difference in the heterogeneity among these eligible articles (*I*^2^ = 84%, *P* < 0.01), so we chose the random effects model. The total pooled effect showed no significant difference between the induction and expectant management (*OR* = 1.38, 95% CI: 0.84–2.28).

### Sensitivity Analysis, Publication Bias, and Risk Analysis

**Figure 3:**
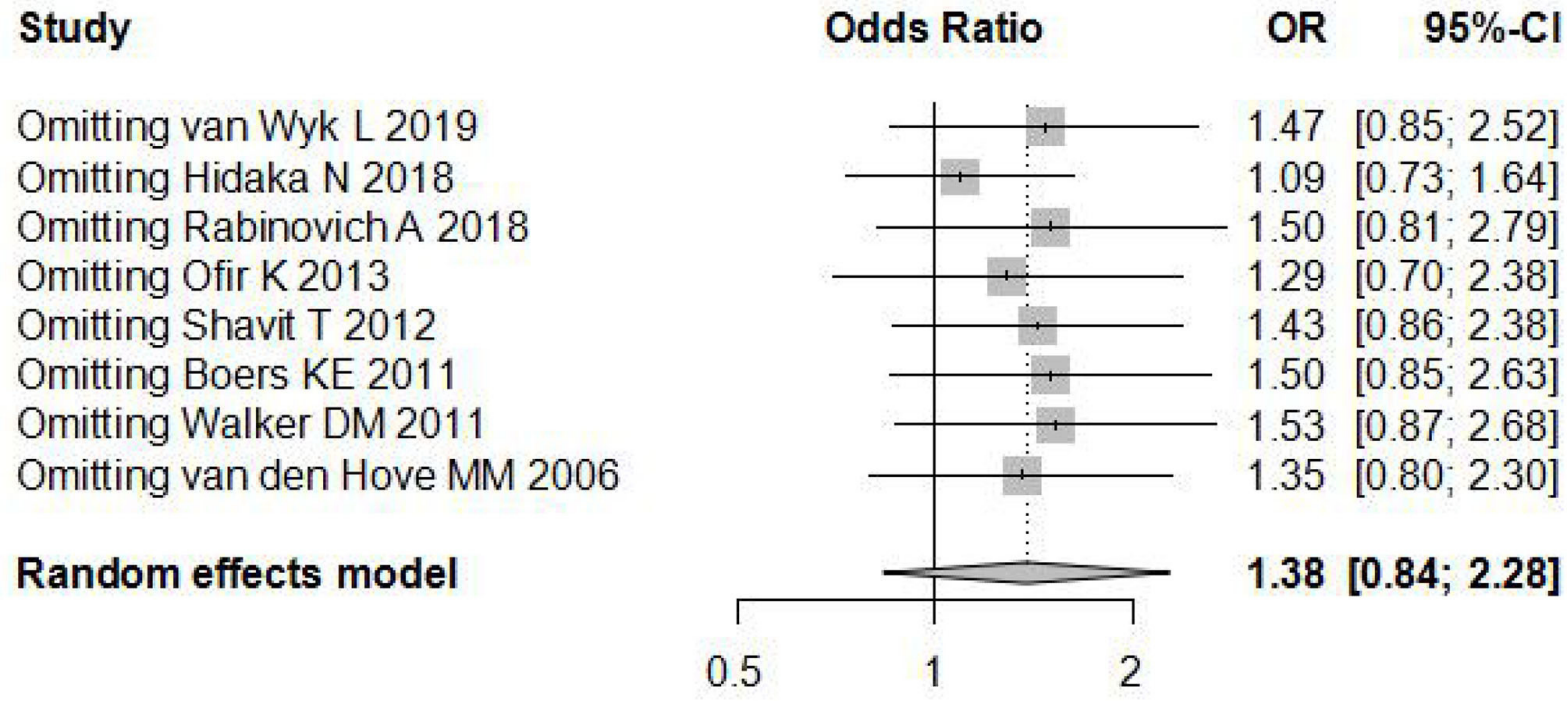
Sensitivity analysis of the included studies.

**Figure 4:**
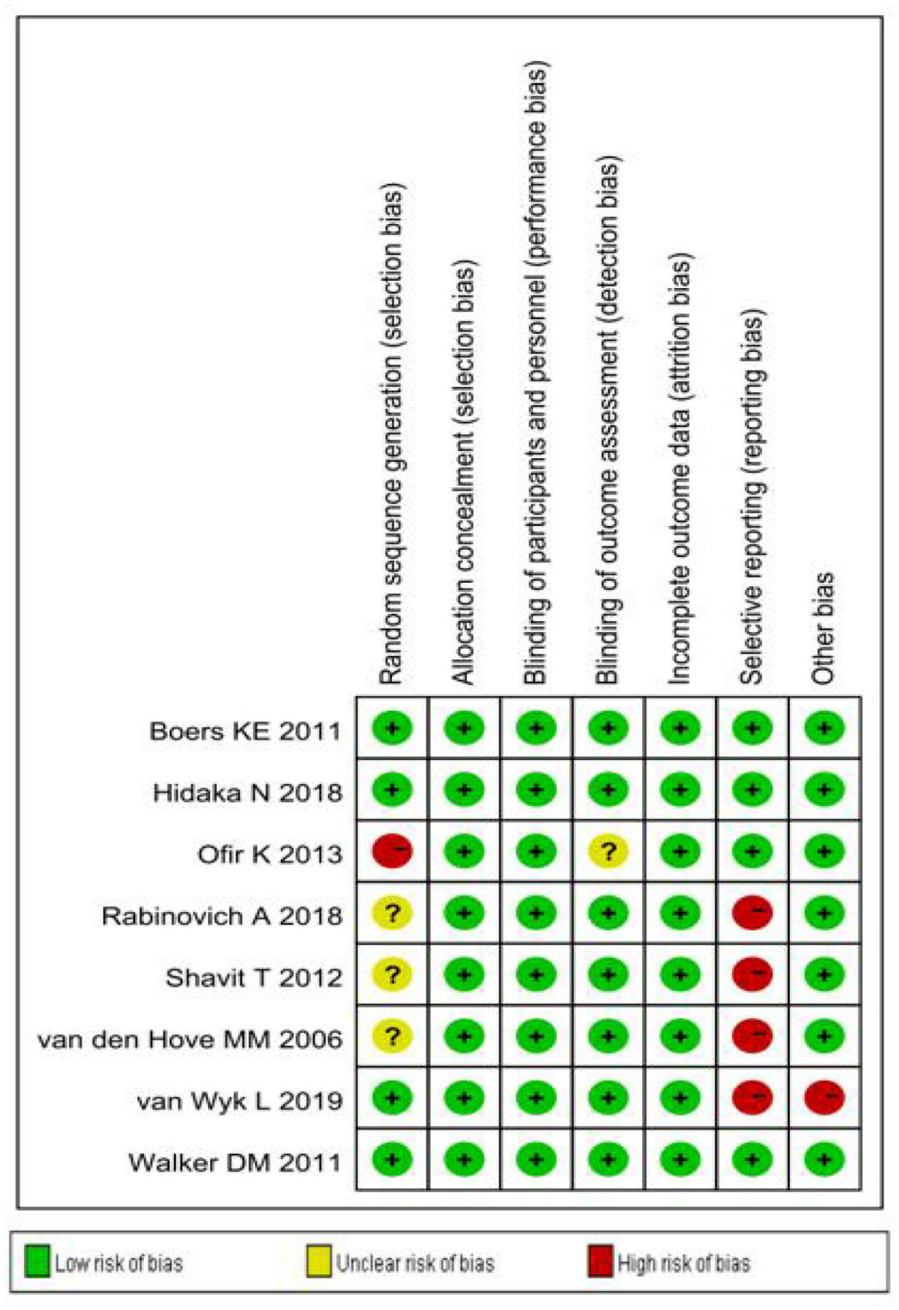
Risk analysis of the included studies.

**Figure 5:**
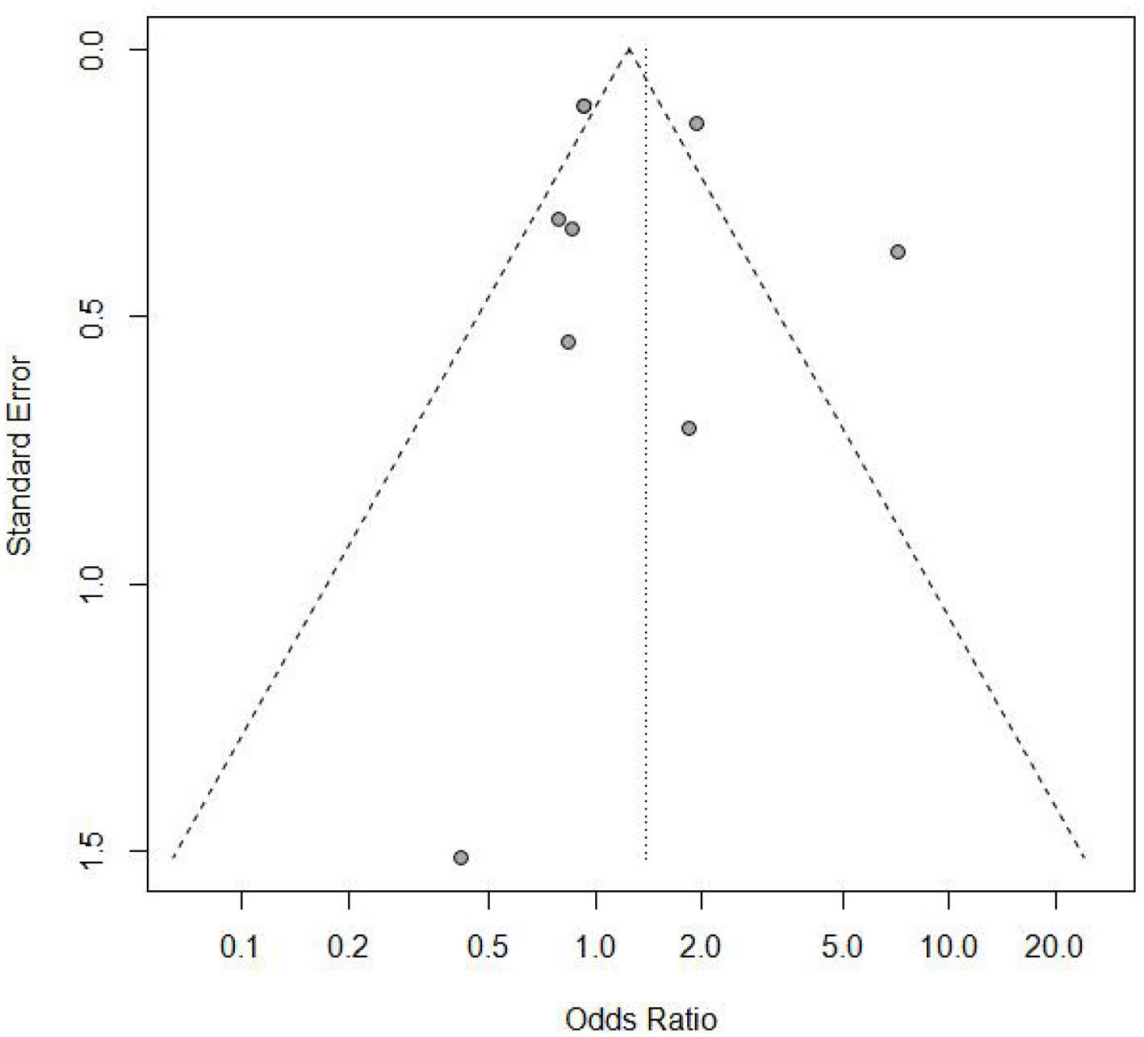
Funnel plot of the included studies.

Sensitivity analysis (Figure 3) showed that when omitting one of these studies_vanWykL,2019_, the *OR* = 1.47, 95% CI: 0.85–2.52, which presents a similar result compared to the total pooled effect (*OR* = 1.38, 95% CI: 0.84–2.28). According to the same way, when omitting any one of the other studies, the neonatal adverse outcomes of induction vs. expectant management still presented the robust result of no statistical significance. The risk analysis of each included study is shown in Figure 4. All eight studies met the criteria of a score ≥5 and the main source of bias was selective reporting. It is difficult to judge whether the cases are representative in the retrospective studies, which is an important reason for the poor score for selective reporting. Additionally, three retrospective studies and one prospective cohort study had problems regarding the randomization of patients and in the prospective study, blind methods were not effectively applied and may have affected the outcome of the study. Thus, we applied the funnel plot to describe the publication bias (Figure 5). The image is basically symmetrical, which indicates that there is no obvious publication bias. In addition, we further evaluated the publication bias by the linear regression equation and found no significant bias (*P* = 0.75).

### Subgroup Analysis

**Figure 6:**
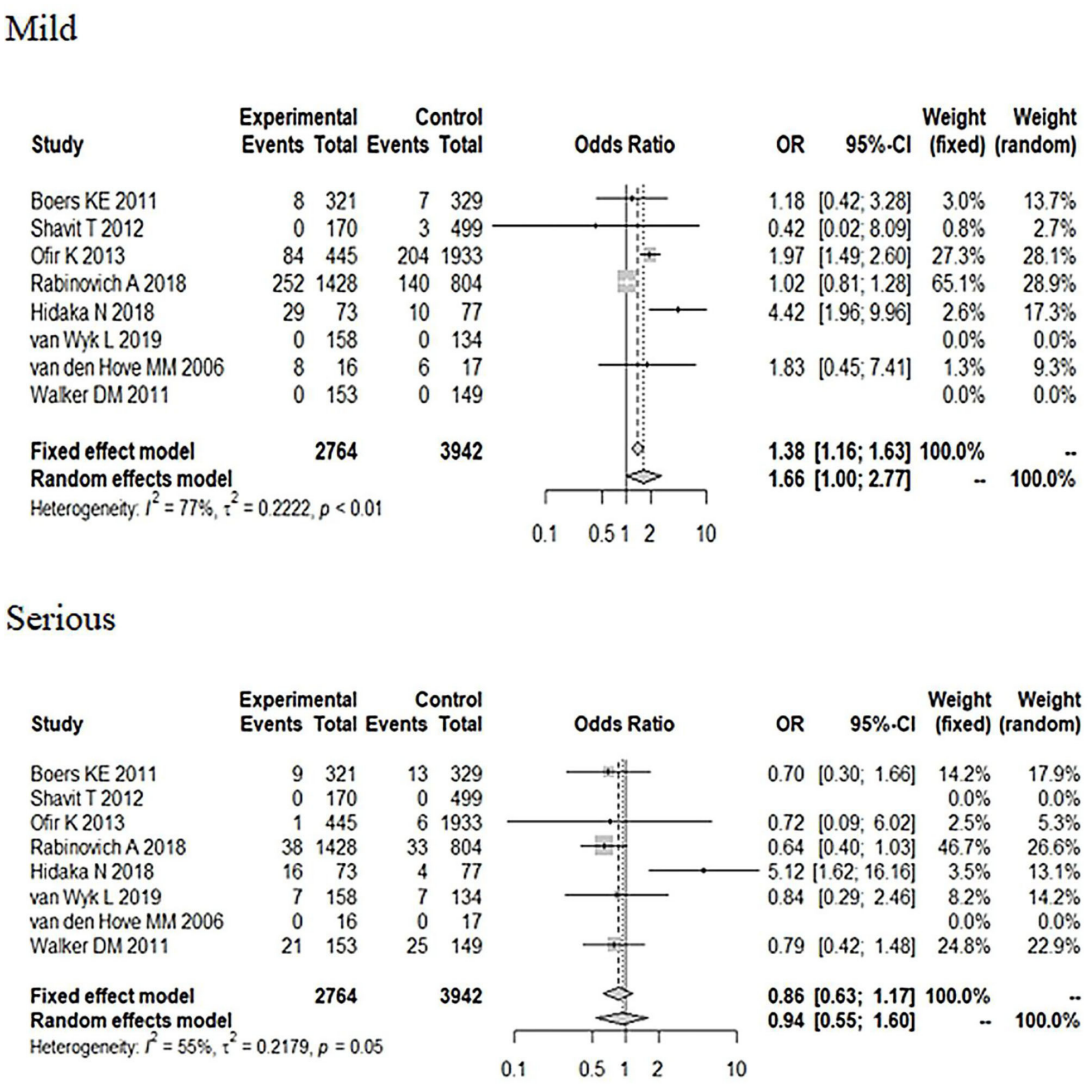
Subgroup analysis of different severity in neonatal adverse outcomes (induction vs. expectant management).

**Figure 7:**
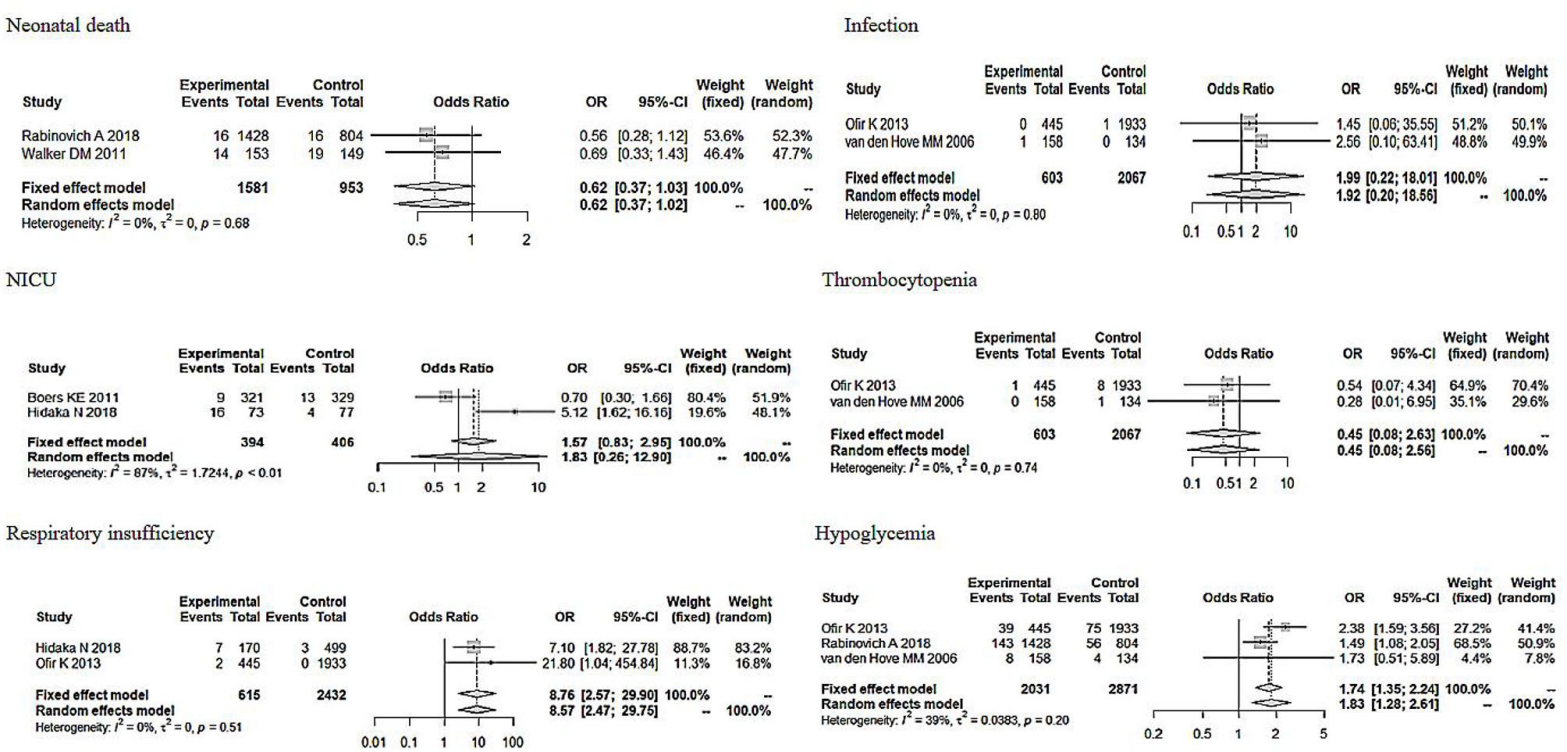
Subgroup analysis of neonatal adverse outcomes (induction vs. expectant management).

We selected the study methods, sample size (≤500, >500), area, NOS score (≤7, >7) Apgar score <7 at 5 min, definition of a suspected FGR, severity, and neonatal adverse outcomes to conduct the subgroup analysis. From Table 2, the subgroup analysis results are nearly consistent with the total pooled effect. Only the heterogeneity based on the Netherlands and NOS score ≤7 obviously changed (*I*^2^ = 0%, *P* < 0.01), and the heterogeneity in the neonatal adverse outcomes of the two studies that did not include the Apgar score <7 at 5 min changed (*I*^2^ = 14%, *P* = 0.28). However, neither effect was significant (OR_Netherlands_ = 0.96, 95% CI: 0.57–1.61; OR_NOSscore ≤7_ = 0.92, 95% CI: 0.76–1.12; OR_barring_ _Apgarscore <7 at 5min_ = 0.91, 95% CI: 0.52–1.61). In regard to the severity of the neonatal adverse outcomes, we created a classification of mild and serious. Neonatal death, NICU, disability, necrotizing enterocolitis, neurological complications, neonatal sepsis, and stillbirth were regarded as serious adverse outcomes, and other outcomes were regarded as mild adverse outcomes (28, 29). As Figure 6 shows, although the heterogeneity showed a change, the total pooled effect still presented no significant difference (OR_mild_ = 1.66, 95% CI: 1.00–2.77; OR_serious_ = 0.94, 95% CI: 0.55–1.60). Through the analysis of different forms of neonatal adverse outcomes, we extracted the outcomes that occurred in more than two studies and conducted a subgroup analysis. The results (Figure 7) indicated that the induction increased the risk of neonatal hypoglycaemia and respiratory insufficiency complications (OR_neonatal hypoglycaemia_ = 8.76, 95% CI: 2.57–29.90; OR_respiratory insufficiency_ = 1.74, 95% CI: 1.35–2.24, respectively).

**Table 2:** Subgroup analysis of neonatal adverse outcomes (induction vs. expectant management).

**Subgroup**	**Studies (*N*)**	**Induction vs. expectant management (*N*)**	**Neonatal adverse outcomes (induction vs. expectant management, *N*)**	***I*^**2**^**	***OR* (95% CI)**
**Study method**					
RCT	4	1,077 vs. 2,545	130 vs. 262	73%	1.10 (0.62–1.96)
Cohort study	4	1,687 vs. 1,397	343 vs. 196	89%	1.83 (0.47–7.11)
**Area**					
Netherlands	3	495 vs. 480	32 vs. 33	0%	0.96 (0.57–1.61)
Israel	3	2,043 vs. 3,236	375 vs. 386	89%	1.26 (0.63–2.51)
Japan	1	73 vs. 77	45 vs. 14	-
The United Kingdom	1	153 vs. 149	21 vs. 25	-
**Sample size**					
≤500	4	400 vs. 377	81 vs. 52	86%	1.74 (0.52–1.83)
>500	4	2,364 vs. 3,565	392 vs. 406	84%	1.15 (0.66–2.00)
**NOS score**					
≤7	5	1,925 vs. 1,603	326 vs. 214	0%	0.92 (0.76–1.12)
>7	3	839 vs. 2,339	147 vs. 244	89%	2.24 (0.87–5.77)
**Apgar score <7 at 5 min**					
Yes	6	2,595 vs. 3,776	444 vs. 427	88%	1.49 (0.82–2.73)
No	2	169 vs. 166	29 vs. 31	14%	0.91 (0.52–1.61)
**Definition of suspected FGR**					
AC or EFW <10th centile for GA	6	2,538 vs. 3,716	407 vs. 419	74%	1.16 (0.73–1.84)
EFW ≤ 1.5 SD for GA	1	73 vs. 77	45 vs. 14	-
UA Doppler waveform is abnormal	1	153 vs. 149	21 vs. 25	-

*RCT, randomized controlled trial; N, number; OR, odds ratio; CI, confidence interval*.

## Discussion

This is the first systematic review and meta-analysis to comprehensively compare the neonatal adverse outcomes between induction and expectant management of a suspected FGR. Eight articles were included in this meta-analysis. According to existing studies and the description above, this meta-analysis indicated that regardless of choosing induction or expectant management, the neonatal adverse outcomes showed no obvious differences. Only when we discuss adverse outcomes separately through the subgroup analysis, which suggested that compared to expectant management, induction may increase the neonatal adverse outcome risks of hypoglycemia and respiratory insufficiency.

Previous studies indicated that early-term (37–38 w) newborns had a higher incidence of complications than late-term (>38 w) newborns (30–32). One of the retrospective cohort studies included 30,229 full-term newborns and showed that the early-term newborns were at a higher risk for respiratory insufficiency, hypoglycemia, and jaundice (30). Another retrospective cohort study concluded that the early-term neonates had an obviously higher risk for hypoglycemia and NICU (31). Our subgroup analysis also suggested that compared to expectant management, induction may increase the neonatal adverse outcome risks of hypoglycemia and respiratory insufficiency. It may be that induction leaves the fetus without enough time to adjust to being removed from the mother's relatively high-sugar environment and unable to deliver enough hormones. A large retrospective cohort study included 120,000 growth restricted fetuses delivered between 36 and 42 weeks, studied FGR management, and timing of the delivery. The results indicated that one more week expectant management for 36–37 w presented less mortality (33). For these fetuses, prolonged gestational age and conservative treatment may be beneficial to them. For pathological growth restriction, the intrauterine environment is not suitable for the fetus to continue to grow in the mother's body. If the gestational age is prolonged, it may increase the risks of hypoxia, acidosis, and even death (11, 12), and immediate induction can help the fetuses outside of adverse growth environments, but no evidence has verified the effectiveness and safety (34). In contrast, studies have reported that induction complications included the overcontraction of the uterus with an abnormal fetal heart rate, leading to an increased rate of cesarean section (CS), fetal distress, uterine rupture, placental abruption, and amniotic fluid embolism, etc. (35, 36). Additionally, several studies have shown that the fetuses with a suspected FGR spontaneously delivered at term did not show increased rates of neonatal adverse outcomes (37–39). Nevertheless, the immediate induction for fetuses with constitutional smallness also has the risk of neurological complications and respiratory insufficiency (11, 12). The study we included could not rule out the effect of pre-term delivery on birth outcomes in unrestricted fetuses, which would have an impact on the final analysis. In addition, in the eight studies, the delivery modes in the FGR induction vs. expectant management groups included a selective CS and spontaneous or involuntary vaginal delivery. Newborns born via a vaginal delivery have more time to adapt to the external environment and can secrete more catecholamines, which is important for lung function (40, 41). Additionally, some studies have reported that the rate of neonatal respiratory insufficiency was higher following a selective CS group than a vaginal delivery (40–44).

Most of these studies focused on short-term morbidity, only a few researchers have explored the long-term consequences between the induction and expectant management of FGR. One prospective study showed that newborns born *via* expectant management in a suspected FGR were more severely growth restricted at birth than those born by immediate induction (21). FGR is a risk factor for cardiovascular disease in children later in life (45–47), however, it remains unclear whether it is caused by a catch-up growth or the potential pathophysiological effect of FGR itself, or both play important roles (21).

There are also some limitations in our study. First we only included eight articles and the limited amount of research may have affected the results. Furthermore, pregnancy is a complex physiological and pathological process and many factors will affect the outcome of the newborn. In our study, the definition of a suspected FGR is inconsistent. Most research defined a suspected FGR as AC or EFW <10th centile or flattening of the growth curve for GA, however, one research defined an abnormal UA Doppler waveform as a suspected FGR and one research used EFW ≤1.5 SD for GA. In addition, there were differences in the gestational age of each included studies, the gestational age in these studies range from 24 to 41 w. Inclusion/exclusion criteria, delivery methods, and adverse outcomes of newborn infants, all of which may have affected the results. On the basis of existing research, there is no sufficient evidence to establish clinical practice guidelines for a suspected FGR at full-term or near-term regarding the benefits from induction or expectant management. In other words, the difficulty lies on how to identify the pathological growth restriction or fetuses with constitutional smallness, how to select the delivery method, and how to select the gestational age for pregnancy termination.

## Conclusion

In conclusion, the results of our systematic review and meta-analysis showed that there is no statistical significance in the adverse neonatal outcomes between induction and expectant management of a suspected FGR in late pre-term infants and full-term newborns. However, our subgroup analysis, which discussed the adverse outcomes separately, suggested that compared to the expectant management, induction may increase the neonatal adverse outcome risks of hypoglycemia and respiratory insufficiency. Pregnancy is affected by many factors, as we described above. In addition, we only included eight articles and the limited amount of research may have affected the results. Future studies should be performed and these additional studies should take the gestational age, delivery method, inclusion/exclusion criterion, and other factors into consideration to exclude the potential confounding factors. The most reasonable way to solve a suspected FGR depends on the specific situation. It is hoped that effective guidelines could be established for the management and treatment of suspected FGR to reduce the incidence of neonatal adverse outcomes.

## Data Availability Statement

All datasets presented in this study are included in the article/Supplementary Material.

## Author Contributions

TL, YW, ZM, XY, and YL developed article ideas, wrote the manuscript, data collection, and analysis. KX and HD reviewed and revised charts and articles. All authors contributed to the article and approved the submitted version.

## Conflict of Interest

The authors declare that the research was conducted in the absence of any commercial or financial relationships that could be construed as a potential conflict of interest.
