# Supplementary material for: Neonatal Adverse Outcomes of Induction and Expectant Management in Fetal Growth Restriction: A Systematic Review and Meta-Analysis
Source: Front Pediatr. 2020 Oct 30;8:558000. doi: 10.3389/fped.2020.558000 (PMC7673389; doi:10.3389/fped.2020.558000)

**Search results**

MeSH term: Fetal Growth Retardation; Entry terms: Intrauterine Growth Retardation; Growth Retardation, Intrauterine; Intrauterine Growth Restriction; Fetal Growth Restriction; [Infant, Small for Gestational Age](https://www.ncbi.nlm.nih.gov/mesh/68007236).

MeSH term: Labor, Induced; Entry terms: Induction of Labor; Labor Inductions; Induced Labor; Labor Induced; Induced, Labor; Labor Induction; Induction, Labor; Inductions, Labor.

Keywords: expectant management; expectant monitoring

Pubmed:


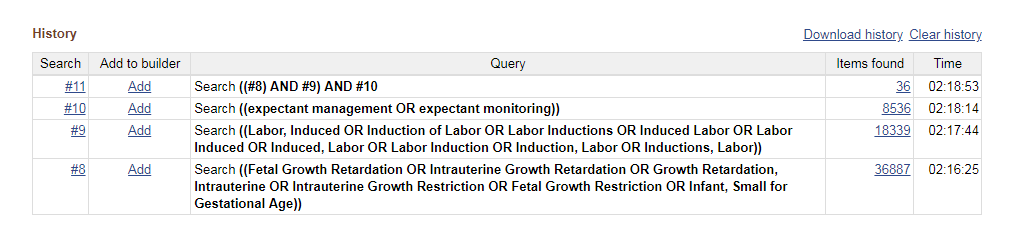


Cochrane Library:


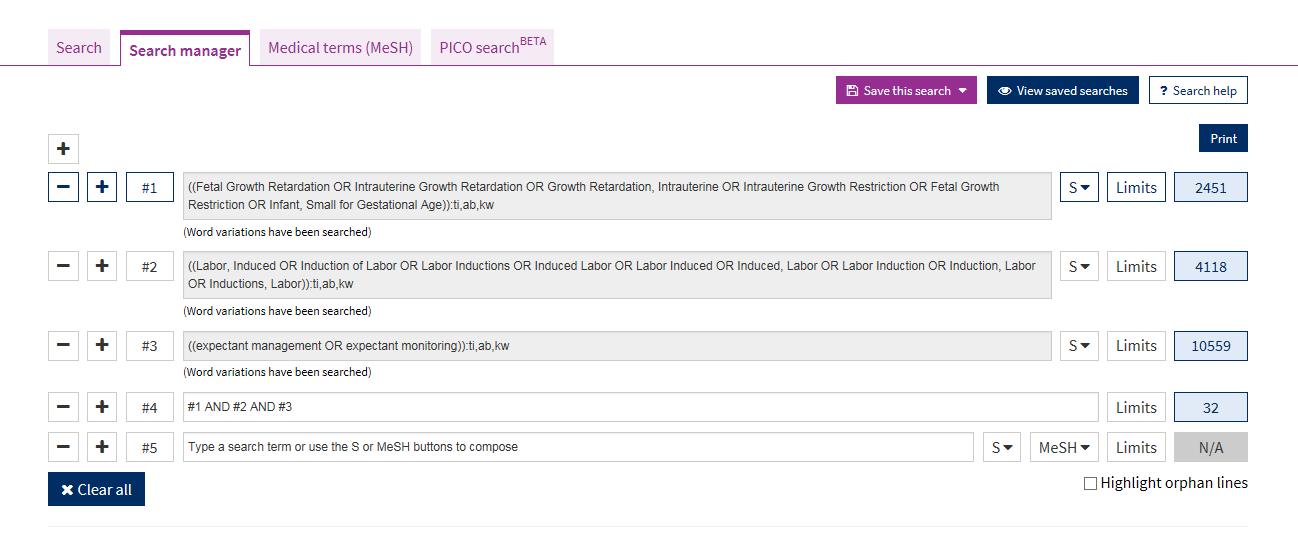


Web of Science:


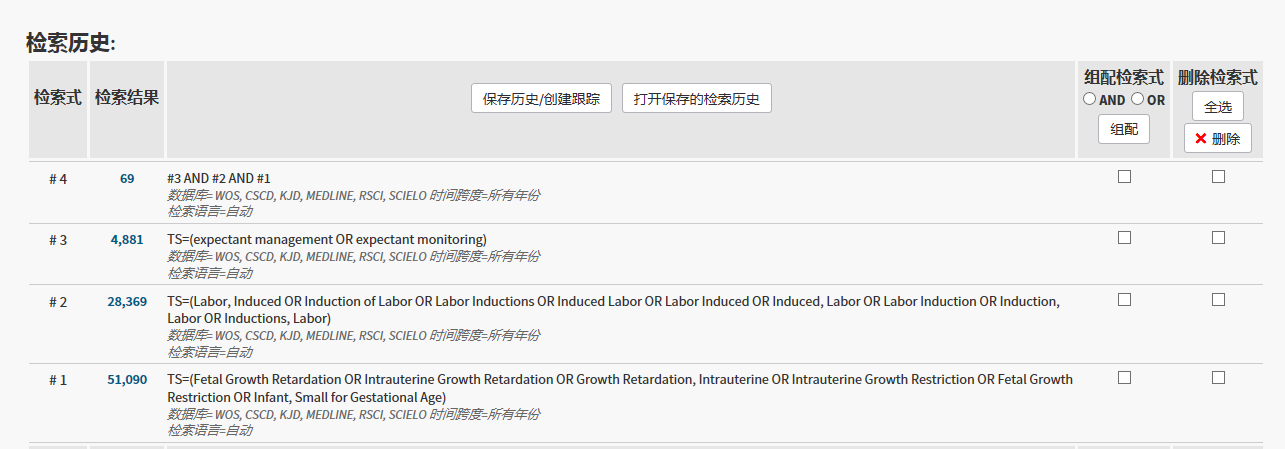

Supplement: Supplementary file 1 [file Table_1.DOCX]
